# Supplementary material for: Mitochondrial genome of Garcinia mangostana L. variety Mesta
Source: Sci Rep. 2022 Jun 8;12:9480. doi: 10.1038/s41598-022-13706-z (PMC9177603; doi:10.1038/s41598-022-13706-z)
Supplement: Supplementary file 1 — Supplementary Figures. [file 41598_2022_13706_MOESM1_ESM.docx]

**Complete Mitochondrial Genome of *Garcinia mangostana* L. variety Mesta**

Ching-Ching Wee^1,2^, Nor Azlan Nor Muhammad^1^, Vijay Kumar Subbiah^2^, Masanori Arita^3^, Yasukazu Nakamura ^3^, Hoe-Han Goh^1*^

**Supplementary Figures**

**Figure S1.** Schematic representation of large and small contigs of Mesta mitogenome.

**Figure S2.** Dot plot analysis showing scf7180000000010 as a circular contig.

**Figure S3**. Mesta mitogenome PacBio read depth.

**
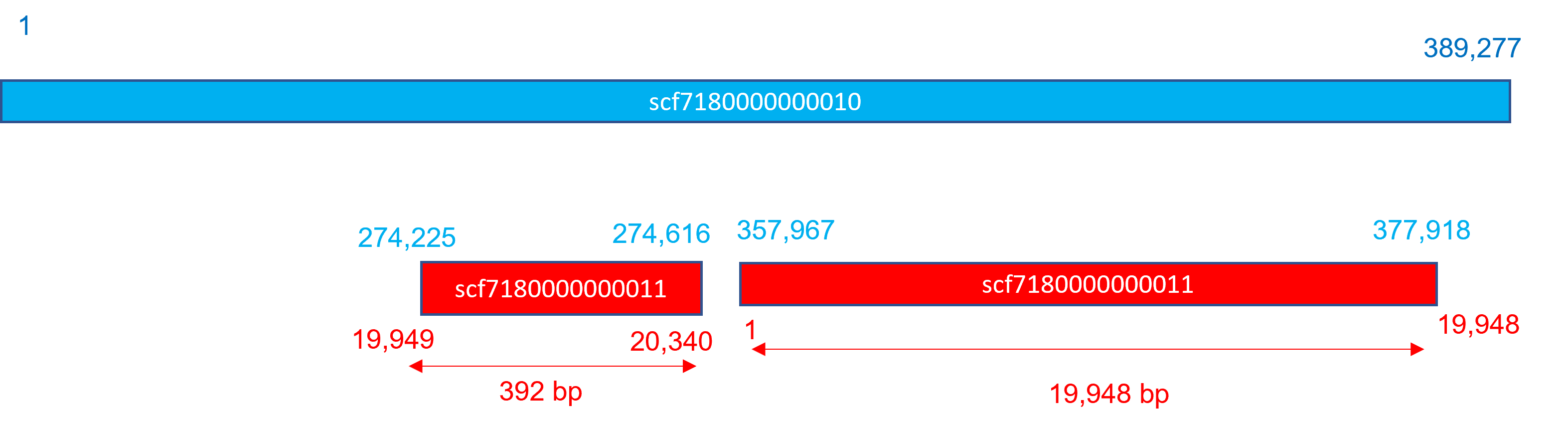
**

**Figure S1.** Schematic representation of large and small contigs of Mesta mitogenome.


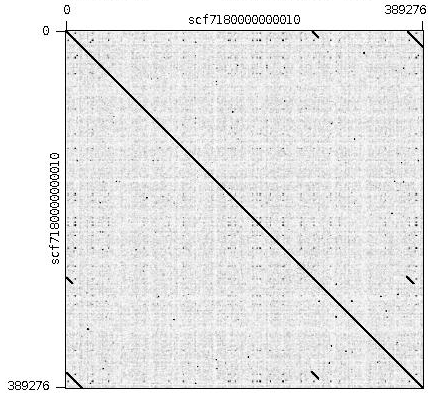


**Figure S2.** Dot plot analysis showing scf7180000000010 as a circular contig.


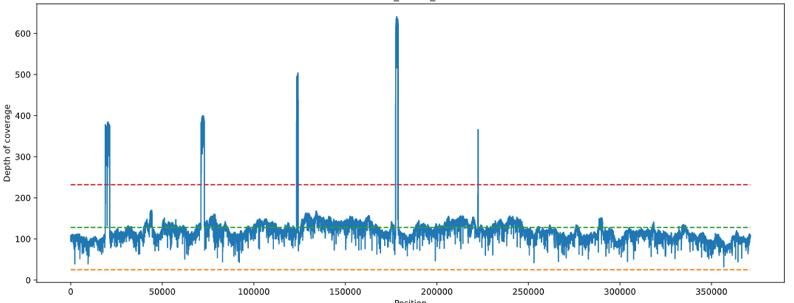


**Figure S3**. Mesta mitogenome PacBio read depth.
